# The Education of Hospital Officers

**Published:** 1915-03-06

**Authors:** John Hy. Johnson

**Affiliations:** Secretary. Royal Westminster Ophthalmic Hospital, W.C.


### THE EDITOR'S LETTER-BOX.

### The Education of Hospital Officers.

*To the Editor of* The Hospital.

Sir,—Your remarks upon Mr. Watts' letter are of great interest to hospital workers, and the question is one that I should like to see widely discussed in your columns. The future of the junior man on the secretarial staff is not a very bright one. His pay is small, and his prospects of promotion slight, as vacancies for senior posts are few and far between. With regard to the recent appointments, I quite agree with Mr. Watts that no doubt the committees had a very good reason in making the selection they did in their appointment of secretary. Might I suggest it was personality?

It has been noticed that a candidate for a secretaryship eminently suited for the position by his long and often varied experience is passed over simply because he did not impress the committee, and to his astonishment sees another man step in whose experience is practically nil. I admit this is discouraging; but until the status of a hospital officer is placed upon a surer and sounder basis those appointments are likely to continue.

With regard to the recently instituted educational scheme of the Incorporated Association of Hospital Officers, it is too early yet to form an opinion as to its utility. Although I have been a member for some years, I have come to the conclusion that it is hardly known to members of committee generally; in fact, with the exception of a few energetic men, its existence is not recognised.

In concluding my remarks, I think the King Edward's Hospital Fund would be the best authority to take the matter up, as they appear to have the welfare of the hospital officer at heart, having made a welcome move in the way of a pension scheme.—I am, Sir, yours faithfully,

John Hy. Johnson, Secretary.

Royal Westminster Ophthalmic Hospital, W.C.
March 1, 1915.

